# High Glucose Increases DNA Damage and Elevates the Expression of Multiple DDR Genes

**DOI:** 10.3390/genes14010144

**Published:** 2023-01-05

**Authors:** Mai A. Rahmoon, Reem A. Elghaish, Aya A. Ibrahim, Zina Alaswad, Mohamed Z. Gad, Sherif F. El-Khamisy, Menattallah Elserafy

**Affiliations:** 1 Center for Genomics, Helmy Institute for Medical Sciences, Zewail City of Science and Technology, Giza 12578, Egypt; 2 Department of Pharmaceutical Biology, Faculty of Pharmacy and Biotechnology, German University in Cairo, Cairo 11835, Egypt; 3 University of Science and Technology, Zewail City of Science and Technology, Giza 12578, Egypt; 4 Department of Biochemistry, Faculty of Pharmacy and Biotechnology, German University in Cairo, Cairo 11835, Egypt; 5 The Healthy Lifespan Institute and Institute of Neuroscience, School of Bioscience, University of Sheffield, Sheffield S10 2TN, UK; 6 The Institute of Cancer Therapeutics, University of Bradford, Bradford BD7 1 DP, UK

**Keywords:** DNA damage response (DDR), hyperglycemia (HG), DNA damage, metabolic diseases, cancer, diabetes mellitus

## Abstract

The DNA Damage Response (DDR) pathways sense DNA damage and coordinate robust DNA repair and bypass mechanisms. A series of repair proteins are recruited depending on the type of breaks and lesions to ensure overall survival. An increase in glucose levels was shown to induce genome instability, yet the links between DDR and glucose are still not well investigated. In this study, we aimed to identify dysregulation in the transcriptome of normal and cancerous breast cell lines upon changing glucose levels. We first performed bioinformatics analysis using a microarray dataset containing the triple-negative breast cancer (TNBC) MDA-MB-231 and the normal human mammary epithelium MCF10A cell lines grown in high glucose (HG) or in the presence of the glycolysis inhibitor 2-deoxyglucose (2DG). Interestingly, multiple DDR genes were significantly upregulated in both cell lines grown in HG. In the wet lab, we remarkably found that HG results in severe DNA damage to TNBC cells as observed using the comet assay. In addition, several DDR genes were confirmed to be upregulated using qPCR analysis in the same cell line. Our results propose a strong need for DDR pathways in the presence of HG to oppose the severe DNA damage induced in cells.

## 1. Introduction

The DNA damage response (DDR) is a series of controlled, complex protein pathways that cells have evolved to ensure genomic integrity. This pivotal signaling explains how cells preserve their function following damage caused by base alteration, aberrant DNA protein function, oxidation or exogenous genotoxic factors [1,2]. The DDR is robustly activated in response to DNA damage, which allows sufficient time for specified DNA repair pathways to physically remove damage [3]. The major DNA repair pathways—base excision repair (BER), nucleotide excision repair (NER), mismatch repair (MMR), homologous recombination (HR) and non-homologous end joining (NHEJ)—are activated throughout different stages of the cell cycle, allowing cells to repair the DNA damage [4,5]. This is in addition to pathways that deal with specific lesions or tolerate them [5,6]. Programmed cell death or apoptosis is activated when the damage persists, which removes cells with extensive genome instability [5].

Many physiological events depend on the DDR network’s coordination of DNA breaks/repair [1]. Deviations in this fine-tuning are known to destabilize cellular metabolic homeostasis, as exemplified in diverse cancers and many metabolic diseases such as diabetes mellitus, where disruption or deregulation of DNA repair pathways results in genome instability [7,8,9]. High glucose (HG) was reported to enhance the cell’s mutation rate and slow the repair ability. This leads to increasing the cell’s susceptibility to oxidative DNA damage [10,11,12,13] and disruption of DNA integrity [14,15,16,17]. Furthermore, the CHK1-mediated DNA damage response is not activated properly in HG conditions [17]. In addition, endogenously induced chemical modifications and adducts in the DNA were reported to be increased due to high glucose metabolism in diabetic models. Metabolic diseases also elevate the circulating glucose levels, resulting in the accumulation of DNA-advanced glycation end products that increase the rate of G transversions. Eventually, these products lead to instability of the genome and a high risk of cancer [18]. For instance, HG levels interfere with the removal of the guanosine derivative produced by the breakdown of glucose: N2-(1-carboxyethyl)-2’-deoxyguanosine (CEdG). HG also destabilizes Hypoxia-inducible factor 1-alpha (HIF1α), which activates several genes involved in the DNA repair process such as the NER genes [19]. This promotes genomic instability and increases cancer susceptibility in people with Type 2 diabetes (T2D) [20,21,22]. An increase in γH2AX protein expression corresponding to DNA breaks was also noticed in high glucose conditions [12,23]. These defects lead to an accumulation of mutations due to the interference of the error-prone NHEJ repair mechanism [24], which is consistent with the negative effects of high glucose and the correlation between diabetes and cancer.

**Figure 1:**
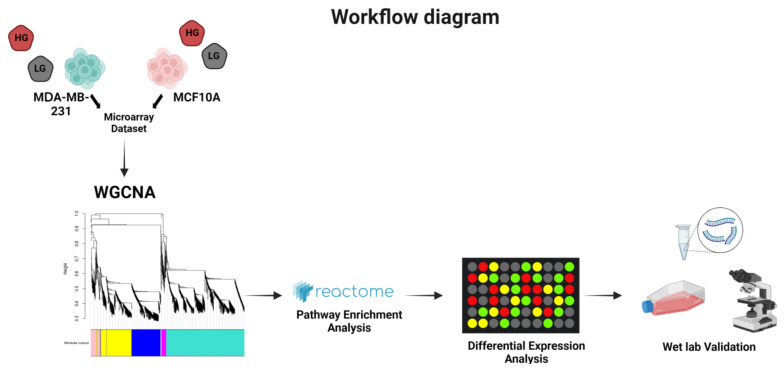
Schematic representation of the workflow for this project “Created with BiRender.com”.

The relationship between HG and DNA damage response/repair mechanisms is still understudied. Therefore, in this research article, we used the triple negative breast cancer (TNBC) cell line MDA-MB-231 and the non-malignant breast cancer cell line MCF10A as a model to analyze the effects of HG on DNA damage and highlight the specific DDR genes that are affected. This work was executed as presented in Figure 1. Weighted Gene Co-expression Network Construction Analysis (WGCNA) was carried out to find the clusters (modules) of highly correlated genes and relate the modules to glucose status of the samples. Then, the Reactome tool was utilized to find the enriched pathways in the selected modules [25]. Differential expression analysis (DEA) highlighted changes in transcription levels in the presence and absence of 2-deoxyglucose (2DG), a widely used competitive inhibitor of glucose uptake and metabolism acting at the level of hexokinase [26]. We then shifted to the wet lab to study a representative group of genes and confirm the findings.

## 2. Materials and Methods

### 2.1. Data Retrieval

The gene expression profiles for mammary cell lines (GSE59228) were downloaded from the Gene Expression Omnibus database (https://www.ncbi.nlm.nih.gov/geo/, accessed on 6 August 2021). The dataset was composed of 8 MCF10A and 8 MDA-MB-231 samples; 4 of each sample type were grown at the low confluence in standard growth conditions and the other 4 were incubated for 24 h with 2-deoxy-glucose (2-DG, 50 mM) to inhibit glucose metabolism.

### 2.2. Data Pre-Processing

The Affy package (version 1.72.0) was used to perform background correction and quantile normalization of the raw .CEL files using the robust multiarray average algorithm (RMA) [27]. After that, the Avereps function implemented in the limma package (version 3.50.3) was used to summarize the expression of the multiple probes for the same gene [28]. To decrease the number of genes for WGCNA, the varFilter function in the gene filter package (version 1.76.0) was used to obtain genes exhibiting the most variation (top 50%) in the expression levels across samples [29].

### 2.3. Weighted Gene Co-Expression Analysis (WGCNA)

The R package WGCNA (version 1.71) was used to perform the weighted correlation network analysis [30]. Firstly, the gene co-expression similarity between genes m and n was defined as Smn = |cor(m, n)|. Given that the scale-free topology was less than 0.8 due to the nature of heterogeneity of the data, the signed network was built using power equals (18) to tackle the high variation between the different cell lines of MCF10A and MDA-MB-231 [30]. Finally, the adjacency matrix was transformed into a topological overlap matrix, and the dynamic tree cut method was also used to identify the modules with hierarchical clustering of the genes using TOM as the distance measure with a deep split value of 2 and minimum module size of 30. Additionally, the corresponding gene information for each module was extracted for further analysis.

### 2.4. Establishment of Module Related Trait Relationships

After the identification of the modules, the module eigengene (ME) was summarized using the first principal component of the module expression levels. Pearson’s correlation analysis was used to identify the association between each individual module and the different phenotypes of high- and low-glucose MDA-MB-231 and MCF10 cells. Each phenotype has a strongly related module which can be considered as its signature [30].

### 2.5. Pathway Enrichment Analysis

The ReactomePA R package was used to identify the enriched pathway based on the Reactome database. The EnrichPathway function utilized a hypergeometric model to evaluate if the number of selected genes involved in a Reactome pathway is larger than expected to evaluate the significance of the enrichment. The cutoff for the adjusted *p*-value was <0.05 [31]. The Enrichplot (version1.14.2) R package was used to visualize the enriched pathways in both the green and brown modules. The tree plot function was utilized to perform hierarchal clustering in a more holistic approach [32].

### 2.6. Differential Gene Expression Analysis

Differential expression analysis was performed using the Limma package (version 3.50.3) [28]. The adjusted *p*-values (adj *p*-value) were implemented to avoid the occurrence of false-positive results. Genes with |log2 fold change (FC)| larger than 1 and adj *p*-value < 0.05 were considered as DEGs.

### 2.7. Cell Culture

The MDA-231 cell line was cultured in DMEM supplemented with 4.5 g/L Glucose (High Glucose) or 1 g/L Glucose (Low Glucose), L-Glutamine, 1% Penicillin/Streptomycin and 10% Fetal Bovine Serum (FBS). The high-glucose medium (25 mM glucose, HG) mimics hyperglycemia in cancer cells, and the low-glucose medium (5.55 mM glucose, LG) is close to the normal physiological conditions of 4–8 mM [33,34].

### 2.8. Alkaline Comet Assay

The alkaline comet assay procedure was executed as described in [35,36]. The Comet Assay IV software (Perceptive Instruments, Suffolk, UK) was used to calculate the tail moment.

### 2.9. Quantitative Real-Time Polymerase Chain Reaction (qPCR)

For the gene expression analyses using qPCR, RNA was extracted from MDA-MB-231 cells using TRIzol. mRNAs from the total extracted RNA were reverse transcribed into the single stranded cDNA using the iScript™ cDNA synthesis kit (Bio-Rad, Hercules, CA, USA) according to manufacturer’s instructions and stored at −80 °C. Quantitative real-time polymerase chain reaction (qPCR) was performed using SYBR™ Select Master Mix (Thermo Fisher Scientific, Waltham, MA, USA) on the QuantStudio™ 12K Flex Real-Time PCR System (Applied Biosystems™, Waltham, MA, USA). Glyceraldehydes-3-phosphate dehydrogenase (GAPDH) was used as a housekeeping gene.

## 3. Results

To explore new possible links between glucose metabolism and changes in gene transcription, we performed bioinformatics analysis using the publicly available GSE59228 microarray dataset. This dataset includes gene expression data for the MCF10A immortalized mammary epithelial cells and the MDA-MB-231 metastatic breast cancer cells cultured in high-glucose media (HG) (25 mM) +/− 2-deoxyglucose (2-DG, 50 mM); an inhibitor of glucose uptake that mimics low glucose conditions [26].

### 3.1. Identification of Key Modules Using WGCNA in Cells Cultured in HG +/− 2-DG

**Figure 2:**
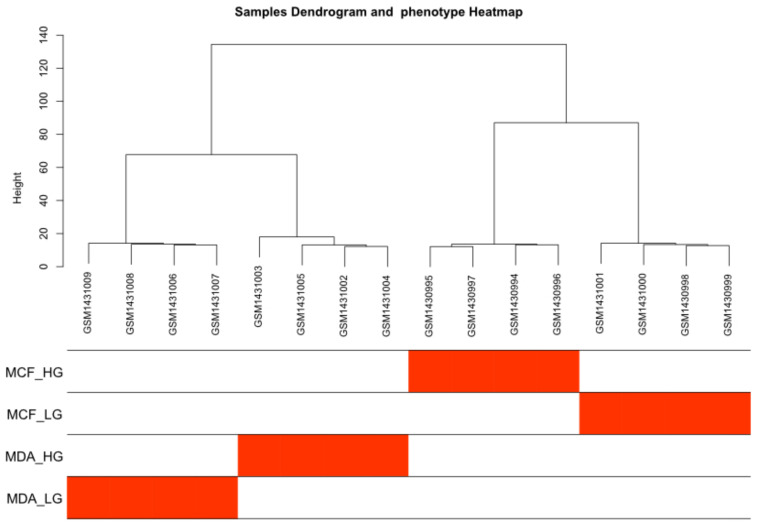
Hierarchical clustering of the samples analyzed. HG, high glucose; LG, 50 mM 2-DG added to inhibit glucose uptake.

The GSE59228 dataset used for the analysis included 16 samples, 8 MDA-MB-231 and 8 MCF10A. Four samples from each type were cultured in high glucose and the remaining four were treated with 2-DG. Firstly, the average link method was used to cluster the samples. The 16 samples were clustered into 2 large clusters. Then, each MDA-MB-23 and MCF10A large cluster was divided into two small clusters according to their glucose level, high glucose (HG) or HG + 2-DG (LG), as shown in Figure 2. According to the average link method, samples from the same cluster exhibit related expressions across all genes. Consequently, the two different cell lines clustered away from each other, and then each cell line clustered into two sub-clusters according to their glucose conditions, which reveals the effect of the difference in glucose levels on the gene expression levels in both cell types.

**Figure 3:**
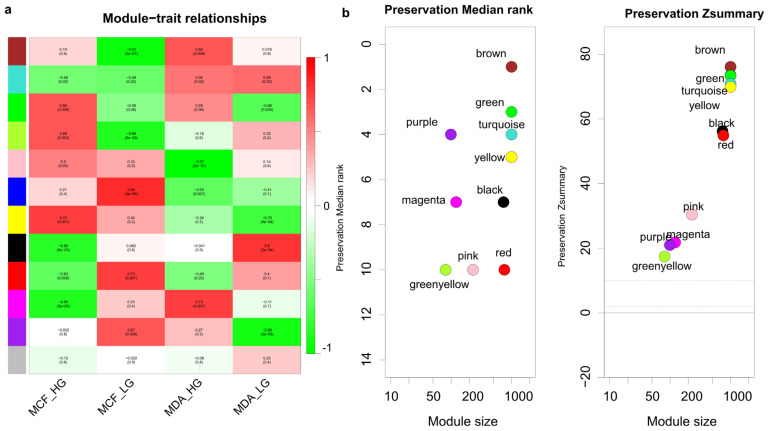
WGCNA analysis to identify the key modules associated with different glucose levels. (**a**) The module–trait relationships were demonstrated using correlation values and *p*-values with a range of colors; the degree of correlation between modules and glucose levels is shown. HG, high glucose; LG, 50 mM 2-DG added to inhibit glucose uptake. (**b**) Median rank and Z-summary statistics in the module preservation tests. Left plot shows the module position in the test dataset based on the median rank. Right plot illustrates the analysis of the Z-summary between different modules.

Using the same dataset, the WGCNA R package was used to cluster genes that are similar in their expression pattern into distinctive modules with the average linkage method. A total of 11 modules were identified (Figure 3a). Then, a module–trait relationship was executed to find the relationship between gene expression profiles and their phenotypes. Pearson’s correlation coefficient was used to identify the association between the module eigengenes and the status of the cells such as the glucose level and the cell type. Furthermore, the *p*-value was calculated for the given correlation in Figure 3a.

The brown and green modules had a significant correlation with the MDA-MD-231 and MCF10A cells in high glucose and their *p*-values were <0.05. The module stability test was used on the same dataset to validate the stability of the identified modules [30,37] (Figure 3b). Since the Z-summary of the brown and green modules was higher than 10 and the median rank was close to the minimum in the test dataset, the modules showed considerable stability. Therefore, we selected these modules for further analysis. The full lists of genes for these two modules are summarized in Tables S1 and S2.

### 3.2. DDR Pathways Are Enriched in the Significant Modules

**Figure 4:**
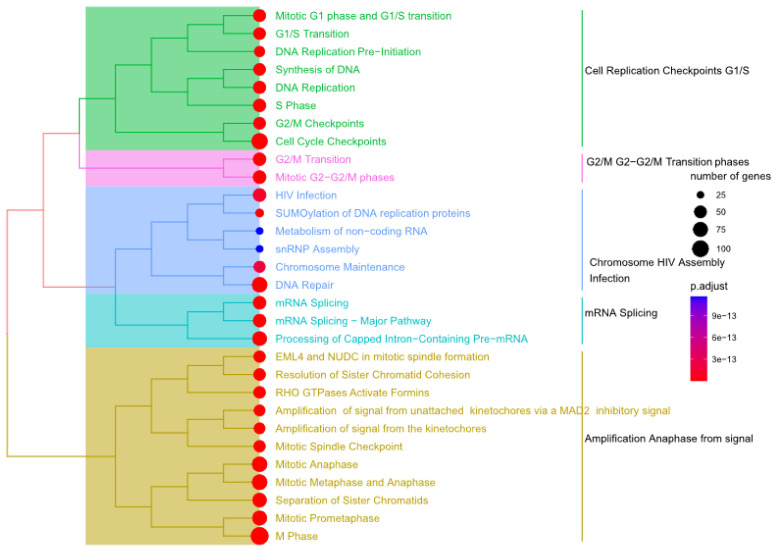
Pathway enrichment analysis for the brown module. The ReactomePA package [38] was used to identify the pathways enriched in the brown modules. The pathways represented are statistically significant (adjusted *p*-value < 0.05) as indicated by the color of the nodes (right panel). The number of genes retrieved in our analysis and identified in the pathways are represented using the size of the circles as indicated in the right panel.

**Figure 5:**
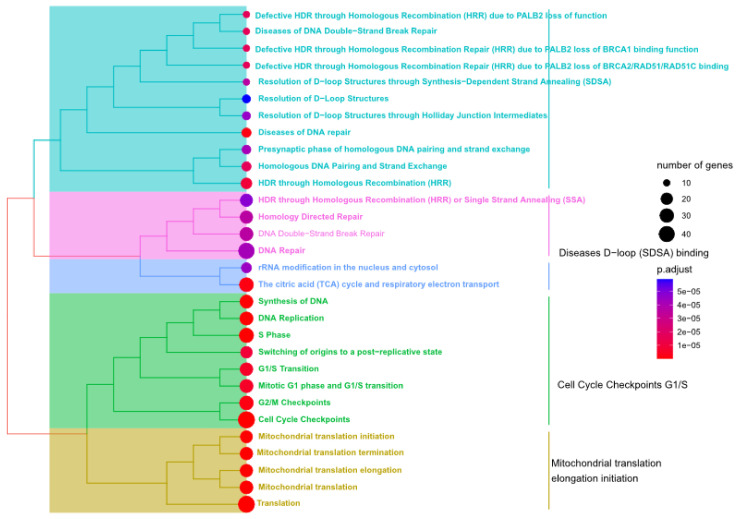
Pathway enrichment analysis for the green module. The Reactome tool [38] was used to identify the pathways enriched in the green modules. The pathways represented are statistically significant (adjusted *p*-value < 0.05) as indicated by the color of the nodes (right panel). The number of genes retrieved in our analysis and identified in the pathways is represented using the size of the circles as indicated in the right panel.

To identify pathways that correlated to fluctuation in glucose levels, the ReactomePA R package (version 3.1.1 March 2017) was used to identify the enriched pathways in the brown (Figure 4) and green modules (Figure 5) [25]. Several DDR pathways were found to be enriched in at least one of the two modules. For example, Homology Directed Repair (HDR), DNA double-stranded break repair, DNA Repair, Cell cycle check points, S-phase, chromosomal maintenance, DNA protein crosslink repair/protein-linked repair, etc. (Figure 4 and Figure 5). The full list of the enriched pathways for the brown and green modules and their significance are summarized in Table S3 and Table S4, respectively.

### 3.3. Multiple DDR Genes Are Differentially Expressed in Cells Cultured in High Glucose vs. Low Glucose

Differential expression analysis (DEA) was completed for the MDA-MB-231 cells and MCF10A cells to identify the differentially expressed genes (DEGs) between the high- and low-glucose status in both cell lines. A *p*-value < 0.05 and the log2 fold change (lfc) >|1| were set as a threshold to identify the DEGs. Interestingly, we observed that multiple DDR genes are down-regulated in LG-cultured cells in comparison to HG cells. Excitingly, the same pattern of expression was observed for numerous genes in both the MDA-MB-231 and MFC10A, indicating that the increase in glucose affects both normal and malignant cells. This is also in line with the WGCNA and the pathway enrichment analysis data. The full list of DEGs for both cell lines is provided in Table S5.

### 3.4. High Glucose Increases the Expression of Multiple DDR Genes Which Is Reversed in Low-Glucose Conditions

**Figure 6:**
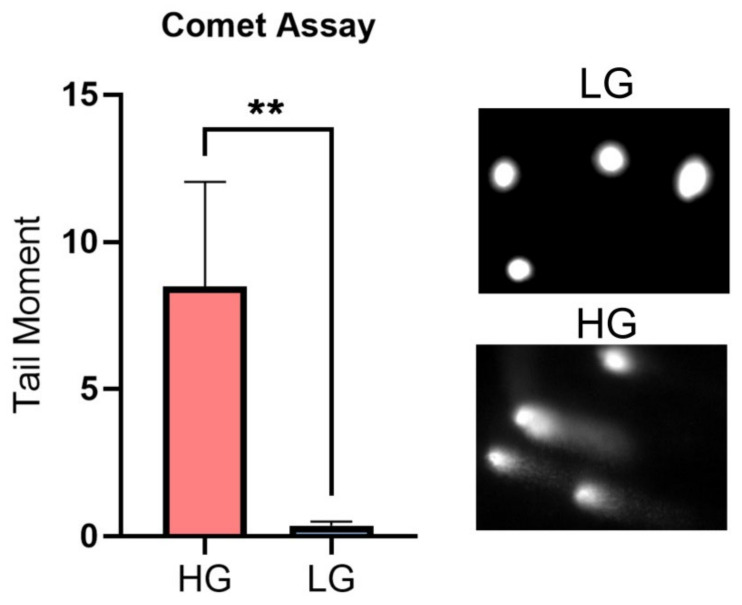
Alkaline comet assay representing significant DNA damage in HG- compared to LG-cultured cells. MDA-MB-231 cells were cultured in high glucose (25 mM glucose, HG) or low glucose (5 mM glucose, LG). Each bar represents the mean ± SEM of three repeats. Results were analyzed using an unpaired Student’s *t*-test where each error bar represents SEM. ** = *p* < 0.01.

To validate the bioinformatics results, we shifted to the wet lab and experimentally exposed MDA-MB-231 cells to LG after culturing in HG. To investigate the effects of high- and low-glucose conditions on DNA damage, a comet assay was performed under alkaline conditions (pH > 13) to detect DNA double-strand breaks, single-strand breaks, alkali-labile sites, DNA-DNA/DNA-protein cross-linking, incomplete excision repair sites and oxidative base alterations [39,40,41,42]. Cells were cultured in high glucose and the comet tail moment was recorded. A higher level of DNA damage was observed in HG cells compared to LG as presented in the increased tail moment (Figure 6).

**Figure 7:**
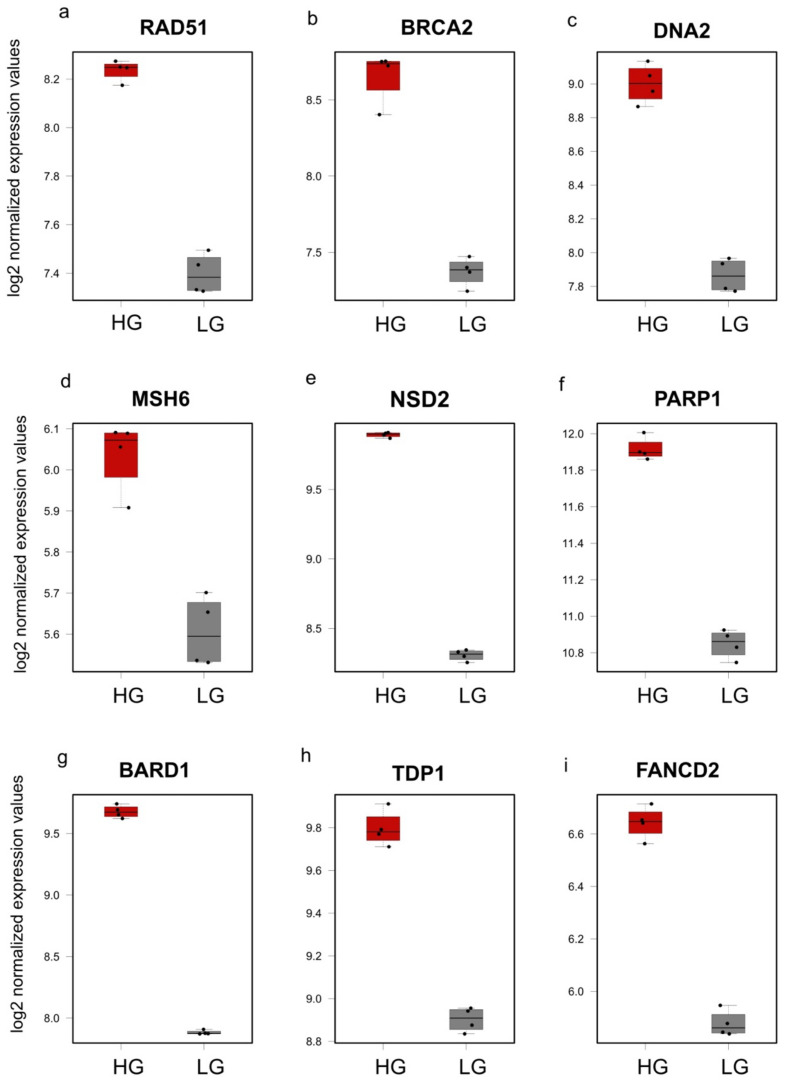
Normalized expression of the differentially expressed DDR genes in MDA-MB-231 analyzed using the microarray dataset. The genes analyzed are represented in (**a**–**i**). Boxplots represent normalized counts in LG (grey) and HG (red). HG, high glucose; LG, 50 mM 2-DG added to inhibit glucose uptake.

**Figure 8:**
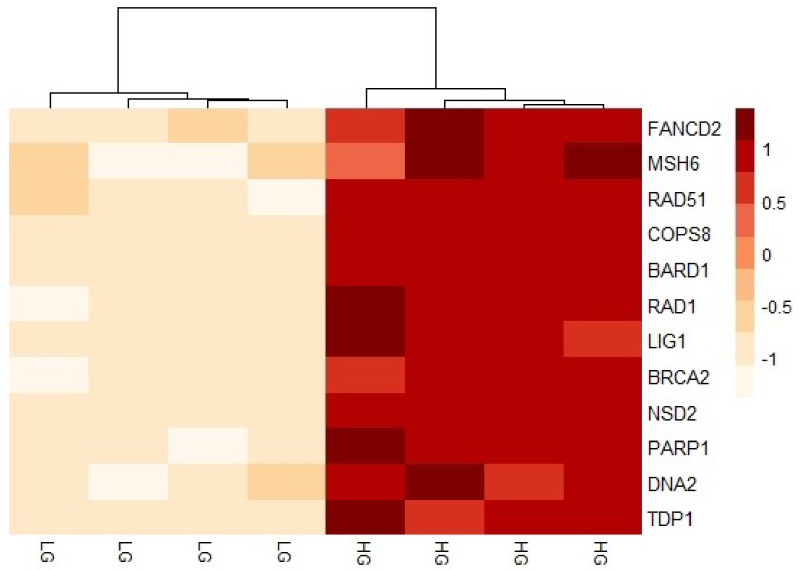
Heatmap of DNA repair genes identified in high- and low-glucose levels in MDA-MB-231 cells. Expression of the genes was normalized and log2 transformed. Both rows and columns are clustered using correlation distance and average linkage. The color intensity reflects the expression levels where positive values indicate upregulation and negative values indicate downregulation. HG, high glucose; LG, 50 mM 2-DG added to inhibit glucose uptake.

**Figure 9:**
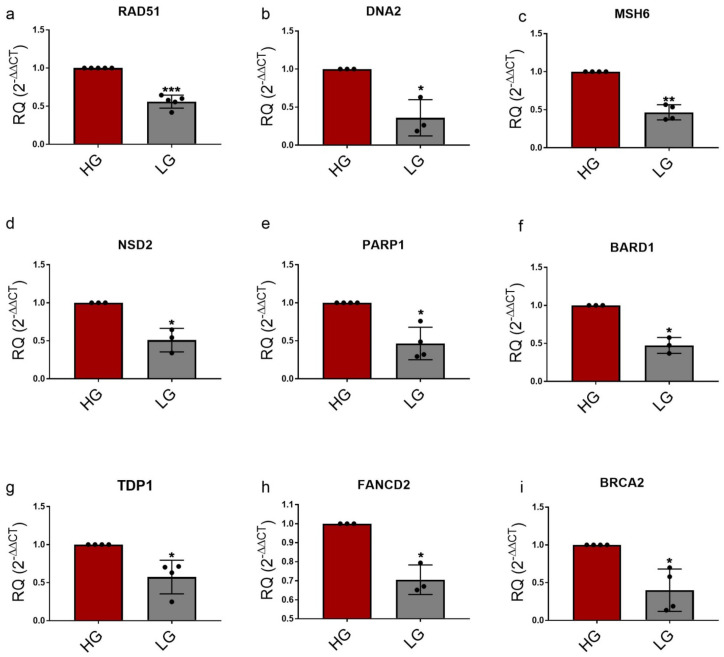
qPCR analysis of DDR genes in HG- vs. LG-treated MDA-MB-231 cells. MDA-MB-231 cells cultured in LG conditions (5.55 mM glucose) resulted in a significant downregulation of genes presented in (**a**–**i**) in comparison to HG (25 mM glucose)-cultured cells. GAPDH was used as a housekeeping gene for the RQ calculations. Data from three or four independent biological replicates of each treatment are presented. Each bar represents the mean ± SEM. Results were analyzed using a paired Student’s *t*-test where *** = *p* < 0.001, ** = *p* < 0.01, * = *p* < 0.05.

To study the effects of changing glucose levels on DDR, we chose representative genes for several DDR pathways for confirmation using qPCR analysis. The genes selected (*BARD1*, *BRCA2*, *COPS8*, *DNA2*, *FANCD2*, *LIG1*, *MSH6*, *NSD2*, *PARP1*, *RAD1*, *RAD51*, *TDP1*) were identified as DEGs and were also part of either the brown or the green modules to cover a broad range of mechanisms (Tables S1, S2 and S5). Moreover, the biological repeats of these genes in the qPCR analysis were consistent and reproducible; therefore, we present the expression levels of these particular genes in the microarray dataset in Figure 7 for MDA-MB-231 and in Figure S1 for MCF-10A. Moreover, to identify major similarities and differences in the transcriptional changes of these genes in the different levels of glucose, we performed hierarchical clustering on normalized expression of DDR genes for each sample. The heatmap shows a clear pattern where the LG and HG cells were clustered together based on the DDR gene expression levels (Figure 8). Finally, the qPCR data is represented in Figure 9 and Figure S2. The Relative quantification (RQ) was calculated for the genes. The data showed that *RAD51* (*p* = 0.0003), *BRCA2* (*p* = 0.0235), *DNA2* (*p* = 0.0431), *FANCD2* (*p* = 0.0223), *MSH6* (*p* = 0.0017), *TDP1* (*p* = 0.0305), *NSD2* (*p* = 0.0318), *PARP1* (*p* = 0.0155) and *BARD1* (*p* = 0.0128) had a significant decrease in expression upon shifting cells to LG in comparison to HG (Figure 9a–i). On the contrary, changes in the *RAD1*, *LIG1* and *COPS8* gene levels were not significantly between HG- and LG-cultured cells in our wet lab experiments despite being differently expressed in the microarray analysis (Figure S2). Overall, the bioinformatics analysis and wet lab experiments showed a great level of concordance, supporting the deleterious effects of HG on genome integrity and cellular functions.

## 4. Discussion

Hyperglycemia promotes oxidative stress and DNA damage, which are significant factors contributing to disease development and progression [43,44]. Since the discovery of the Warburg effect over a century ago, the role of glucose in cancer formation and progression has drawn substantial attention. Hyperglycemia results in an increased prevalence and mortality associated with many cancers, including breast and colorectal [45,46,47,48,49]. It was reported that HG significantly increases mutations in phosphoribosyltransferase and thymidine kinase loci in human lymphoblastoid cell lines and Lac1 in the mouse embryo, thus affecting genomic stability [13,50,51]. In addition, it causes DNA lesions and strand breaks and alters the DNA damage response in renal and prostate cancers [17,52]. Chemo- and radiation-resistance were also noted in normal renal epithelial cells and renal cell carcinoma after high glucose exposure and attributed to altered DNA damage response and reduced repair, though DNA repair protein expression changes were not examined [17]. Additionally, alterations in the *XRCC1* gene and protein expression were reported following glucose concentration changes in breast cancer cell lines and hepatocytes [53,54]. XRCC1 is an essential protein in DNA repair and is known to be involved in the single-strand break (SSB) and base excision repair (BER) pathways. Particularly, XRCC1 is recruited to the repair of oxidative DNA breaks by BER pathways [55]. It was also found that high glucose exposure drives *XRCC1* expression through increased STAT3 activation, resulting in resistance to DNA damaging agents [56]. A recent report also observed that the expression of γH2AX protein, which corresponds to DNA double-strand breaks, was increased in high-glucose conditions [17]. Despite previous efforts establishing a link between hyperglycemia and DNA damage, the impact of high-glucose concentration on DNA repair genes is understudied [17].

In this article, bioinformatics analysis of a microarray dataset of normal and malignant breast cell lines, MCF10A and MDA-MB-231, respectively, was used to study the impact of high glucose on DDR pathways. WGCNA followed by pathway enrichment analysis revealed an over-representation of DNA damage response pathways upon changing glucose levels. We also interestingly detected significant DNA damage as DNA double-strand breaks, single-strand breaks, alkali-labile sites, DNA-DNA/DNA-protein cross-linking, incomplete excision repair sites and oxidative base alterations using the alkaline comet assay [39,40,41,42] in cells cultured in HG, which explains the need for a strong DDR response to control this damage. While the dysfunction of DNA repair proteins through their loss or mutations has gathered significant research focus, factors driving the overexpression of DNA repair proteins, such as hyperglycemia, are not well understood. Therefore, we tried to select specific DDR genes to propose possible consequences on specific cellular pathways in HG conditions.

In our experiments, the most significant upregulated genes included *FANCD2*, a player in Fanconi anemia (FA) [57], and several genes playing a role in the HR-mediated repair of double-strand breaks (DSBs) such as *BARD1*, *BRCA2*, *RAD51* and *DNA2* [58,59]. Other genes identified as DEGs included *TDP1*, which catalyzes the excision of stalled topoisomerase I-DNA complex [60,61,62,63,64], the mismatch repair gene *MSH6* [65,66] and the Poly (ADP-ribose) polymerase-1 (*PARP1*) that mediates multiple repair pathways [67,68]. Most of the genes analyzed produced consistent results between the microarray analysis and the qPCR analysis. However, some differences were observed, for example, in the *LIG1*, *RAD1* and *COPS8* genes. The minor differences between the expression levels in the bioinformatics analysis and wet lab results can be attributed to different factors. Firstly, despite the usage of the same MDA-MB-231 cell line, some variations might exist between different labs [69]. In addition, increasing the number of clones analyzed in the microarray analysis would produce better results. Performing RNA-Sequencing could even produce more solid results [70]. However, overall, both the bioinformatics analysis and the wet lab data greatly support an elevation in DDR genes in the presence of high glucose.

**Figure 10:**
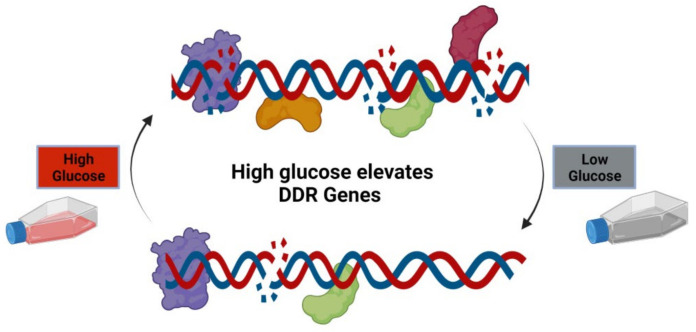
A concluding model showing the HG impact on DNA damage. In the presence of high glucose, the DNA accumulates high damage which requires an increase in the recruitment of DNA damage response genes. This is in contrast to LG conditions that showed a decrease in DNA damage “Created with BioRender.com”.

Our results propose a model where growing cells in HG increases the levels of DNA damage and consequently the expression of DDR genes to fix this damage. Yet, despite this elevation, the damage seems to accumulate in HG-cultured cells to an extent that is not completely repaired by the overexpressed DDR genes/proteins. On the contrary, shifting cells to LG conditions decreases the levels of DNA damage and, accordingly, the need for DDR (Figure 10).

As previously mentioned, hyperglycemia is suggested to have a profound impact on cancer progression. We have also previously observed an upregulation in 36 DDR genes in ductal and lobular breast carcinomas in comparison to normal breast cells [70]. Therefore, we expected that the increase in the expression levels of DDR genes would be specific to the breast cancer cell line (MDA-MB-231). However, to our surprise, MCF10A cells cultured in high glucose resulted in an elevation in DDR gene expression. This indicates that the negative effects of glucose upregulation affect normal cells as well.

Our results provide novel insights into possible consequences on DDR pathways in patients who suffer from uncontrolled diabetes and an increase in blood glucose levels. It also opens new questions regarding the extent of damage caused to normal cells vs. cancer cells. This link may concern the development or progression of cancer as well as whether diabetes’ relationship with cancer may be influenced by cellular reactions to DNA damage. Our findings concur with others that demonstrate elevated levels of DNA damage in diabetic patients’ white blood cells [71,72,73,74]. To put it another way, we can speculate that the increase in blood sugar levels in patients with uncontrolled diabetes may lead to a higher risk of developing cancer [75]. Metabolite-induced DNA damage, DDR and persistent DNA damage signaling are common soil for several complications of diabetes-like cancer. Recognition of this common soil may lead to novel therapies and better treatment modalities for DM-cancer patients. Our findings suggest that measuring the degree of endogenous DNA damage using the comet assay may predict the risk of developing cancer in diabetic patients. Combining the transcriptome analysis of DDR with proteome analysis to confirm that the gene upregulation is directly reflected on the protein level would also produce more accurate conclusions on the status of repair in patients’ cells.

## 5. Conclusions

In this study, we showed that cells exposed to high-glucose conditions manifested an increase in the transcription of multiple DDR genes and exhibit significant levels of DNA damage. These effects were interestingly reversed to a great extent upon exposure of cells to LG conditions (Figure 10). Although we should not hide from the fact that the effect appears cancer-independent, the consequences could be more aggravated and more relevant to cancer cells. Our data provide new insights into the effects of increased sugar levels on DDR and genome stability. This intriguingly raises new questions that can link hyperglycemia and metabolic disorders such as diabetes mellitus to DNA damage and cancer progression.

## Data Availability

The dataset used for the analysis was downloaded from https://www.ncbi.nlm.nih.gov/geo/query/acc.cgi?acc=GSE59228, accessed on 1 October 2022.

## Supplementary Materials

The following supporting information can be downloaded at: https://www.mdpi.com/article/10.3390/genes14010144/s1, Figure S1: Normalized gene expression of the differentially expressed DNA repair genes in MCF10 cells. Boxplots represent normalized expression values in LG (grey) and HG (red); Figure S2: DDR genes that showed no changes in transcription levels using qPCR between HG and LG in MDA-MB-231 cells. The left panel represents the qPCR data and the right panel represents the microarray data. Boxplots represent normalized counts in LG (grey) and HG (red). The GAPDH gene was used as an internal control in all qPCR experiments; Table S1: List of co-expressed genes in the green module; Table S2: List of co-expressed genes in the brown module; Table S3: The full list of the enriched pathways for the brown module; Table S4: The full list of the enriched pathways for the green module; Table S5: The full list of DEGs for the MDA-MB231 and MCF10 lines cultured in HG vs. LG.
