# Supplementary figures and images for: High Glucose Increases DNA Damage and Elevates the Expression of Multiple DDR Genes
Source: Genes (Basel). 2023 Jan 5;14(1):144. doi: 10.3390/genes14010144 (PMC9858638; doi:10.3390/genes14010144)

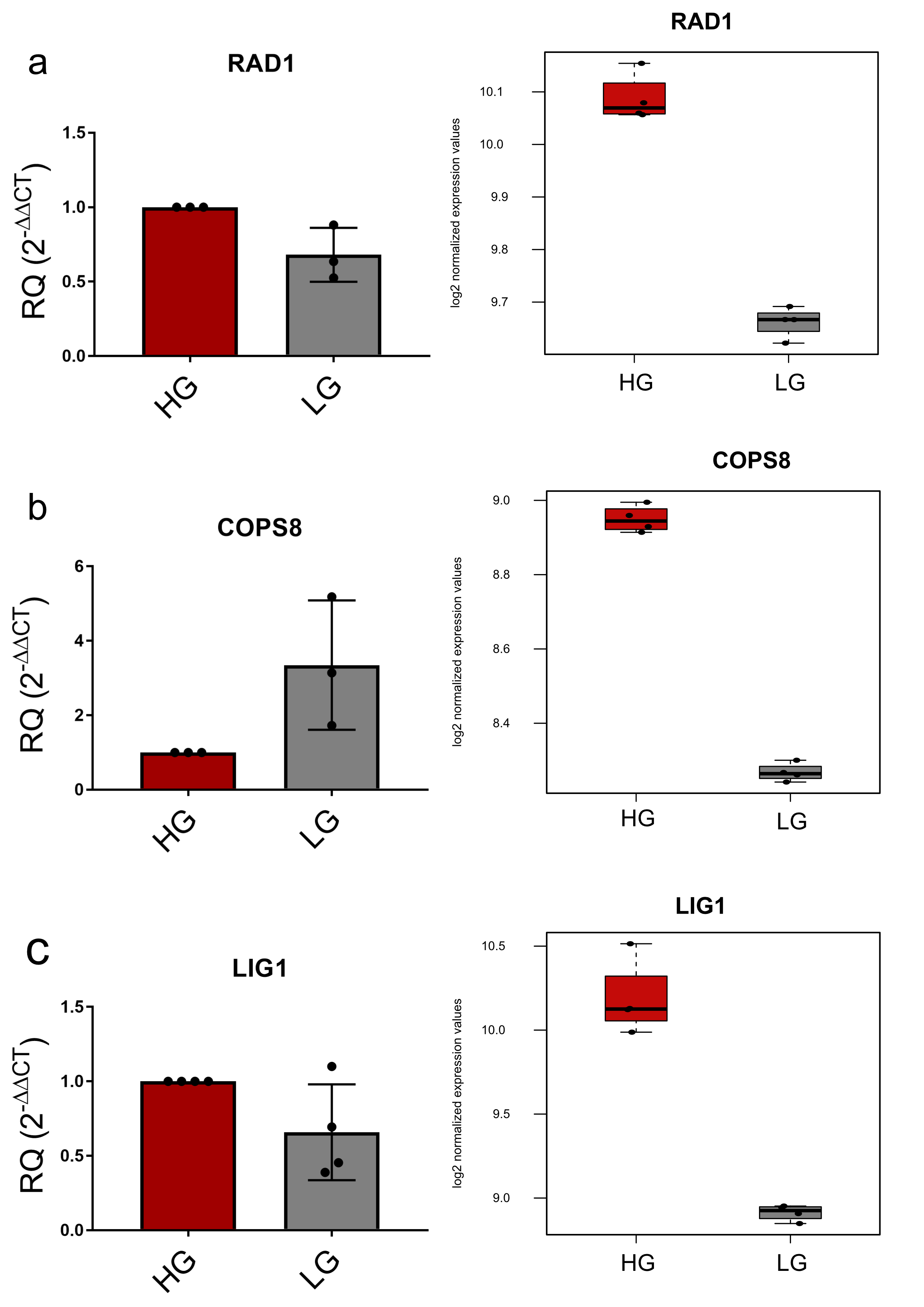

Supplement: Supplementary file 1 [file genes-14-00144-s001.zip › Fig.S2_DDR.jpg]

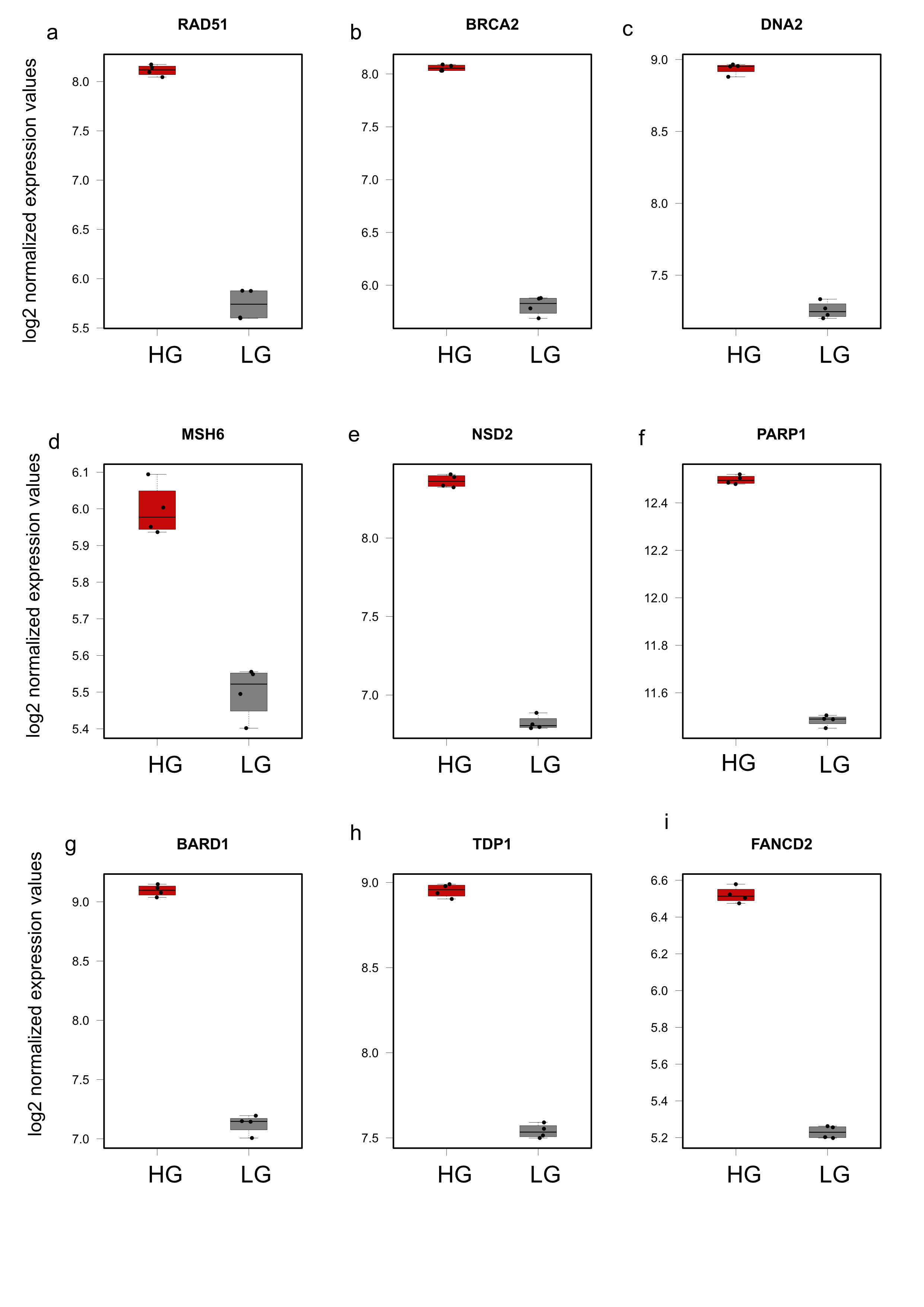

Supplement: Supplementary file 1 [file genes-14-00144-s001.zip › Fig.S1.jpg]
